# Ultrafiltration in Heart Failure: A Review

**DOI:** 10.7759/cureus.39933

**Published:** 2023-06-04

**Authors:** Himanshi Bisht, Apoorva Tripathi, Akshat Arya, Ashwati Konat, Divya Patel, Dhruvin Godhani, Rushi Kamaria, Parita Shah, Gayatri Chudasama, Pragya Jain, Kamal Sharma

**Affiliations:** 1 Medicine, Byramjee Jeejeebhoy (BJ) Medical College, Ahmedabad, IND; 2 Internal Medicine, Byramjee Jeejeebhoy (BJ) Medical College, Ahmedabad, IND; 3 Department of Zoology, Biomedical Technology and Human Genetics, Gujarat University, Ahmedabad, IND; 4 Internal Medicine, Gujarat Medical Education and Research Society (GMERS) Medical College, Gandhinagar, IND; 5 Internal Medicine, Government Medical College, Surat, IND; 6 Internal Medicine, Smt. Nathiba Hargovandas Lakhmichand (NHL) Municipal Medical College, Ahmedabad, IND; 7 Cardiology, Dr. Kamal Sharma Cardiology Clinic, Ahmedabad, IND

**Keywords:** decompensation, fluid overload, heart failure, diuretics, ultrafiltration

## Abstract

Ultrafiltration is an effective method to get rid of fluid retention and congestion in patients with acute decompensated heart failure (HF) without affecting the circulating volume. Although its efficacy in comparison to diuretics is debatable, the evaluation of our analysis is based on various studies that comprise published clinical trials on ultrafiltration and studies comparing the efficacy of diuretics and ultrafiltration. Apart from this, we also look at literature that provides shortcomings of the said procedure and its scope for future advancements. Heart failure ultimately leads to volume overload, which is a highly concerning complication. Diuretics have been used as a first-line treatment for fluid overload but are becoming inefficacious due to the development of resistance and renal dysfunction. Ultrafiltration, on the other hand, is an attractive alternative to counter volume overload and congestion, which are unresponsive to medical therapy. There is also evidence that it significantly decreases the probability of future episodes of decompensation. There are, however, disagreements about whether ultrafiltration is an effective method to improve mortality in these patients. There is a lack of conclusive studies demonstrating the superiority of one fluid removal method over another. Hence, it is imperative to continue searching for the most effective method to treat congestion. Priority should be given to more mechanistic studies regarding ultrafiltration.

## Introduction and background

With a prevalence of more than 23 million globally, heart failure (HF) is a significant public health issue with major concerns such as increased morbidity, mortality, and cost [1,2]. One of the key causes of heart failure is congestion and fluid overload [3]. Fluid retention is a manifestation of compromised natriuretic and renal endocrine reactions to volume expansion, which in turn may lead to renal and hepatic dysfunction [4]. In order to maintain the vital stability of such patients, fluid congestion needs to be actively managed.

Loop diuretics have been the mainstay treatment for managing patients with congestive heart failure [5,6]. Despite the widespread use of diuretics, several adverse events have been associated with their prolonged use, including acute kidney injury, impaired neuro-humoral activation, and electrolyte imbalance [7]. Apart from this, diuretic resistance is another significant concern that seems to negate the pharmacological benefits of diuretics [8]. This warrants the need for an alternative strategy to counter the above-mentioned problems.

Ultrafiltration is one such emerging strategy that promises better management of fluid overload and congestion without the limitations that come with diuretics. It operates on the principle of mechanically removing excessive plasma water from the blood through a semipermeable membrane, with the help of a pump-generated hydrostatic pressure [9]. This allows for progressive fluid overload resolution since the fluid withdrawn is continually replenished by fluid from the third space [10].

Ultrafiltration as a replacement for diuretic therapy is an attractive avenue for research. There are still a lot of factors that need to be taken into consideration before determining how effective it can be. The aim of this review is to highlight the various advantages and disadvantages of ultrafiltration in heart failure and the emerging and unconquered territories in the same.

## Review

Discussion

Pathophysiology

Acute heart failure (AHF) is a clinical illness that frequently necessitates hospitalisation and has the potential to be fatal, with congestion being one of the most significant reasons [3,11]. This condition may be caused by a variety of factors, but the main one is a defective natriuretic and renal endocrine response to acute volume expansion, even in asymptomatic patients [4]. Enhanced central venous pressure, a sign of increased intravascular volume, causes congestion and restricts blood flow in the renal veins, resulting in a net reduction in glomerular filtration, which manifests in the form of acute kidney injury that is referred to as cardiorenal syndrome type 1 [12]. Apart from this, it is also linked to endothelial damage, intestinal ischaemia, and hepatic impairment [13,14]. Taking all this into consideration, decreasing congestion needs to be a primary target for patients with heart failure. Aggressive fluid drainage, however, can result in an increase in creatinine levels, the causes of which are currently unclear, including a decline in glomerular filtration rate (GFR) or acute tubular injury [15].

Diuretics to Counter Heart Failure and Its Consequences

Loop diuretics are drugs that have been used to great effect in patients with heart failure with complications involving congestion. Rapid diuresis brought on by intravenous loop diuretics lessens dyspnea and lung congestion [14]. But frequent exposure to loop diuretics causes their efficacy to diminish [16]. High readmission rates may be influenced by unresolved congestion [17]. Furthermore, loop diuretics may have detrimental effects on neurohormonal activation, electrolyte balance, and cardiac and renal function, which might lead to an increase in morbidity and mortality [18,19]. Loop diuretics most commonly used in patients are furosemide, bumetanide, and torsemide.

Mechanism of Action

Diuresis achieved in patients by the use of diuretics is achieved via blockade of the Na^+^/K^+^/2Cl^-^ co-transporter that is present at the thick ascending loop of the Henle portion of the nephron; this inhibition by loop diuretics is responsible for reducing the Na^+^ in urine and reabsorption of Cl^-^ ions, eventually leading to natriuresis along with diuresis [20]. So as to sustain an euvolemic state of the body, diuretics are usually prescribed at the bare minimum dosages. Diuretics may be given both in the oral form as well as intravenously, but to achieve a faster pharmacodynamic response, they could be used as an intravenous agent in patients with acute decompensated heart failure (ADHF) [21].

Advantages

Traditional clinical practise guidelines with the general consensus that diuretics aid in the relief of congestion symptoms in patients presenting with fluid retention in the management of heart failure have classified diuretic therapy as a "class I" recommendation (evidence and/or general agreement that a given treatment or procedure is beneficial, useful, or effective) [3,22]. Loop diuretics decrease ventricular remodelling and mitral regurgitation when administered in conjunction with vasodilators, increasing cardiac output [23]. Longer hospital admissions and worse mortality have been linked to inadequate symptom alleviation with diuretics, highlighting the significance of efficient decongestion in enhancing ADHF outcomes [24,25]. Diuretics are drugs that can be taken orally as well, contrary to ultrafiltration, wherein a patient is hooked up to a circuit through an intravenous access route. Fundamentally, a patient is said to be more compliant with a course of loop diuretics than with ultrafiltration as a treatment modality taken into consideration in patients with chronic heart failure (CHF).

Disadvantages and Limitations

Diuretics, although having their own set of pros over ultrafiltration, also have a number of limitations regarding their use to counter the effects of congestion in heart failure. Chronic diuretic use is linked to a negative effect (excessive activation) on the neurohormonal pathway, which is linked to an increase in mortality rates in hospitalised patients [26]. As loop diuretics are "threshold medications," which implies that an ample amount of dosage is required to be administered to attain a therapeutic response, high doses of diuretics are giving rise to a new challenge observed in recent history termed diuretic resistance [27,28]. This hampered diuretic response has no particular definition, but in all accordance, it indicates the inefficiency of achieving a therapeutic response, and the exact culprit behind this resistance cannot be narrowed down to a single factor [29,30].

Loop diuretics also cause activation of the sympathetic nervous system and renin-angiotensin-aldosterone system (RAAS), which eventually may lead to electrolyte imbalance (hypokalemic hypochloremic metabolic alkalosis) as a result of worsening renal function (WRF) [31,32].

Ultrafiltration as a Management Strategy

In order to establish ultrafiltration as a reliable management strategy and a promising alternative to diuretic management, we discuss the mechanism of action of ultrafiltration, its certain advantages over diuretics, and the unexplored areas related to its efficacy or demerits.

Mechanism of Action

Ultrafiltration occurs across a semipermeable membrane, also known as a haemofilter. Here, whole blood is filtered across the membrane, which results in the production of plasma water. A transmembrane pressure gradient is a prerequisite here [3]. A single or double-lumen cannula is used to obtain peripheral venous access. A blood pump creates a significant pressure gradient, which is in turn connected to a haemofilter, producing the ultrafiltrate [33]. A set of detectors is also part of this whole ultrafiltration circuit, such as haematocrit (HCT) or air sensors. Blood is eventually returned to the same vein from which it had been withdrawn via the alternate flows after passing through the haemofilter [34]. The advantages of smaller size, portability, low blood flow rates, and the ability to offer a wide range of UF rates (0-500 ml/h) are all provided by the newer, simpler UF devices, which also do not require admission to intensive care units [35].

Advantages Over Diuretics

Various studies and clinical trials have demonstrated certain advantages of ultrafiltration over diuretics. Many of these advantages are attributed to the outflux of fluid from extravascular space, as this improves signs and symptoms of pulmonary oedema, dyspepsia, and orthopnoea. A reduction in pulmonary wedge pressure is also noted. Moreover, these clinical improvements persist for six months after one session of ultrafiltration. Diuresis and cardiac output increase without any noticeable changes in heart rate, systolic blood pressure (SBP), mean arterial blood pressure, or electrolyte levels. Therefore, there is a reduced risk of hypokalaemia and hypomagnesemia [36]. Apart from this, fluid removal from the body can be properly regulated with ultrafiltration. It also removes total body sodium without causing a significant change in the electrolyte distribution. Along with the reversal of diuretic resistance, ultrafiltration is also believed to restore the responsiveness of the patient to diuretics because ultrafiltration does not activate the RAAS or neuro-humoral system as seen in the case of diuretics [37].

Unexplored Areas

In view of the abovementioned advantages, it is evident that ultrafiltration is the most feasible alternative management strategy to diuretics. However, there are various knowledge gaps and unexplored areas as far as ultrafiltration as the mainstay of management is concerned; hence, it calls for more intensive and elaborate discussion and research. Moreover, the selection of patients and target setting for fluid removal are completely misunderstood as far as ultrafiltration is concerned. Owing to the economic infeasibility and potential complications of UF, it cannot be used in all heart failure patients. UF causes considerable alterations in tissue perfusion, reductions in central venous oxygen saturation, skin vasodilation, and prolonged arterial hypoxemia, among other hemodynamic, thermal, and respiratory stresses. Acute renal damage is the main side effect of UF. A large hemodynamic shift brought on by UF fluid removal may result in transient renal hypoperfusion. It is now understood, though, that a brief increase in serum creatinine during AHF medication is not always a sign of an acute kidney injury and may not be a useful indicator of unfavourable long-term results. Infection, flow issues, extracorporeal circuit clotting, bleeding owing to excessive anticoagulation, and hypotension due to intravascular depletion are additional concerns associated with catheter access that may necessitate vasopressor support [38]. The equipment for UF costs about US $25,000, and each use of the supplies costs about US $900. Although ultrafiltration is still one of the more expensive treatment options for ADHF, it has been hypothesised that its effect on decreasing the hospitalisation rate may have long-term financial benefits. If these expenses are mitigated by a shorter hospital stay or a change in how these patients are cared for outside of intensive care, more research is required. There is no available prospective cost analysis of UF, and the little retrospective investigation produced conflicting and unsatisfactory results [39]. There are many hypotheses to be worked on and tested; for instance, it is believed that UF is more effective in patients with urinary sodium concentrations of <100 meq; this mandates a randomised trial [40]. Apart from this, monitoring UF therapy can become a daunting task as the attainment of low cardiac filling pressure is not the final indication of fluid overload remission. Ultrasonography might be needed in these cases [41,42]. Due to the lack of optimal methods for the assessment of blood volume and fluid excess, there is difficulty in monitoring and remission of the patient undergoing ultrafiltration therapy. According to the CARRESS-HF study, approximately 16% of UF cases get terminated due to an increase in serum creatinine. This points towards a correlation between UF and worsening renal function. This unexplored area regarding UF needs to be paid heed [43]. The use of specific biomarkers during UF treatment is another untapped area for further research potential as far as ultrafiltration therapy in heart failure patients is concerned [44].

Clinical Trials

Heart failure requires suitable decongestion, but this may result in volume depletion, which has proven to be a hazardous complication [45]. With ultrafiltration being increasingly considered as a substitute for diuretic therapy, it becomes empirical to weigh the advantages and shortcomings through evidential data. Numerous randomised control trials (RCTs) have been conducted regarding the same and have provided useful insight into ultrafiltration treatment for heart failure.

Patients with CHF were randomly assigned to the unfractionated (UF; n = 20) or standard care (SC; n = 20) groups in the first randomised controlled trial, RAPID-CHF (relief for acutely fluid-overloaded patients with decompensated heart failure), with weight loss being the endpoint [23]. The primary diagnosis of CHF was given to study participants who were hospitalised. They had to have two or more lower extremity oedemas and at least one of the following conditions: ascites, elevated jugular venous pressure of 10 cm H_2_O, pulmonary oedema or pleural effusion on chest X-ray, pulmonary rales, pulmonary wedge, or left ventricular end-diastolic pressure of 20 mm Hg. These patients underwent a single, eight-hour session of UF, with the attending physician determining the fluid removal rates. At the treating physician's discretion, additional courses of UF were permitted, but only after the 24-hour endpoints had been evaluated. The median amount of time from consent to the start of UF was 3.9 hours, while the average length of UF was eight hours per session. 3213 ml was the average amount of ultrafiltrate that was extracted during UF. Four patients (20%) received an extra UF therapy session 24 to 48 hours after giving their assent. In the standard care and UF groups, respectively, the median cumulative dose of furosemide (or similar doses of other IV diuretics) received during the first 24 hours was 160 mg and 80 mg, resulting in an extra 3,650 to 4,175 ml of ultrafiltrate clearance. When compared to usual care, UF caused a considerably greater fluid loss within the first 24 hours (4650 mL vs. 2938 mL, p = 0.001) and a quicker recovery from symptoms. The UF group lost more weight at 24 hours, 2.5 kg versus 1.86 kg in the non-UF group of patients (p = 0.24). Overall, the experiment showed that early UF administration to patients with acute heart failure was well tolerated and may have supplemented routine medical therapy [46].

Another single-centre research study, early ultrafiltration therapy in patients with decompensated heart failure and observed resistance to intervention with diuretic agents (EUPHORIA), looked at 20 ADHF patients who had a fluid overload and diuretic resistance and were taking 80 mg of furosemide, 40 mg of torsemide, or 2 mg of bumetanide daily with more than or equal to 1.5 mg/d of blood creatinine. UF was started within 4.5±3.6 hours in 19 HF PTS with volume overload and creatinine of more than or equal to 1.5 mg/dl before hospitalisation and before IV diuretics. The following describes the patient's characteristics: 74% male, 95% white, 74% ischemic HF, ejection fraction of 31±16%, age 75. The study has shown that extensive fluid evacuation (8500 ml via UF) did not result in hypotension, aberrant electrolytes, or WRF. An intriguing finding of the EUPHORIA research is that only one patient needed hospitalisation in the same period following UF, after nine individuals needed it in the three months before the treatment [47].

In the UNLOAD (ultrafiltration versus intravenous diuretics for patients hospitalised for acute decompensated congestive heart failure) RCT, patients with fluid excess brought on by heart failure were randomly assigned to receive early UF versus normal diuretic therapy. Patients needed to be at least 18 years old, be receiving treatment for heart failure (HF), be assigned within 24 hours of hospitalisation, and meet two of the following criteria for hypervolemia: Jugular venous distension of ≥7 cm; pulmonary rales, paroxysmal nocturnal dyspnea, or orthopnea; radiographic pulmonary oedema or pleural effusion; an enlarged liver, or ascites; or peripheral oedema ≥2+. Ultrafiltration was the only method used to treat hypervolemia for the first 48 hours following enrollment; intravenous diuretics were not allowed. The treating physician sets the time and rate (up to 500 ml/h) of fluid removal. Intravenous diuretics were administered to patients who were randomly assigned to usual care. Weight loss and a dyspnoea assessment 48 hours after randomisation served as the main objectives. Patients in the UF group had lost more weight and net fluid at the time of the outcome. Scores for dyspnoea were comparable between groups. In terms of electrolyte imbalance, deteriorating renal function, hypotension, or bleeding frequency, there were no safety issues associated with UF. Notably, the UF arm had a much lower rate of rehospitalisation at 90 days, indicating that one of the main advantages of UF over diuretics may be the maintenance of euvolemia even after discharge [48].

For the purpose of comparing the clinical, bio-humoral, and hemodynamic effects of the two therapies, the ULTRADISCO (ultrafiltration vs. diuretics on clinical, bio-humoral, and haemodynamic variables in patients with decompensated heart failure) study randomly assigned 30 patients with overload due to decompensated heart failure to either UF or intravenous diuretics [49]. Patients were randomised upon admission following a clinical evaluation and had to be at least 18 years old. Peripheral oedema of 2 or more was required for admission, as well as at least one of the following: Pulmonary crackles or rales; dyspnea, paroxysmal nocturnal dyspnea, orthopnea, or tachypnea; the third heart sound; jugular venous distension; positive hepato-jugular reflux; maximal pulmonary pressure values of >50 mmHg as determined by a two-dimensional echocardiogram; or radiographic pleural effusion. Beginning with a minimum fluid removal rate of 100 ml/h and a maximum of 300 ml/h, ultrafiltration was performed. Systolic arterial blood pressure (SAP) measurements were taken into consideration when adjusting the pace of fluid drainage. Depending on the patient's clinical status, the length of the ultrafiltration treatment varied. Furosemide was continuously infused into patients who had been randomly assigned to receive intravenous diuretic therapy at an initial dose of 250 mg/24 hours. PRAM29, a tool that enables researchers to do non-invasive measures of haemodynamic variables, was used to monitor patients who were divided into UF and standard care groups [31]. N-terminal proBNP (NT-proBNP) and aldosterone were two measures that decreased more in patients receiving UF. The arterial pressure parameters were unaltered during the UF procedure and dramatically dropped following the infusion of diuretics, indicating improved haemodynamic stability with UF treatment. It is interesting to note that the UF group demonstrated a considerable improvement in haemodynamic condition as measured by the stroke volume index, cardiac index, cardiac power output, and a significant decrease in systemic vascular resistance [49].

The CARESS-HF (cardiorenal rescue study in acute decompensated heart failure) sought to evaluate UF's contribution to the treatment of acute heart failure with cardiorenal syndrome. It was the most alarming report to be released to date and cast serious questions on the efficacy and safety of UF. In total, 188 individuals were included in the study, split evenly between the UF and pharmaceutical groups. Inclusion requirements for patients were: decompensated heart failure was the primary diagnosis upon admission to the hospital at age 18 years; the beginning of the cardiorenal syndrome (an increase in creatinine of more than 0.3 mg/dL) during or after hospitalisation or continuous volume overload. Following the insertion of the proper intravenous access, ultrafiltration was started at a fluid removal rate of 200 ml/h and continued until the patient's congestion-related indications and symptoms were minimised. Although there were only insignificant differences in weight loss, the UF group showed a higher increase in blood creatinine levels. Additionally, throughout the course of the 60-day follow-up period, a larger percentage of UF patients experienced major negative consequences like kidney failure, haemorrhage, and central venous catheter-related problems [50]. Major apprehensions were raised about the CARRESS-HF study's methodology and results. First, diuretics were given to 39% of the UF group in place of UF (9%), or they were given diuretics after UF was withdrawn (30%). The UF rate was required to be 200 ml/h for every patient, whereas in the diuretic group, the medication was titrated depending on the urine output. Additionally, 90% of the UF group had not sufficiently decongested by the time the primary endpoint was assessed. The CARRESS-HF trial's key outcomes prompted researchers to carry out a second study in which the UF rate may be altered [47].

The CUORE (continuous ultrafiltration for congestive heart failure) experiment suggests that in patients with overload brought on by congestive heart failure, UF therapy may offer some long-term advantages over conventional diuresis. Age >18 years, New York Heart Association (NYHA) functional class III or IV, left ventricular ejection fraction (LVEF) less than or equal to 40%, and an estimated weight gain of more than or equal to 4 kg due to peripheral fluid overload in the previous two months were the requirements for inclusion. One or two sessions of ultrafiltration were administered to patients in the ultrafiltration group, up to a cumulative fluid removal of >2 litres. In both groups, the decision to administer extra medical treatment was up to the patient's assigned cardiologist. Both groups received the same IV diuretic dosage that was started prior to randomisation, unless the clinical condition called for a change. The treating physician was given complete discretion over the length of each session and the ultrafiltration rate (100-500 ml/h), which might fluctuate depending on the clinical circumstances. The incidence of rehospitalisations for congestive HF in patients receiving ultrafiltration versus conventional treatment was the main outcome measure in the CUORE study. In the ultrafiltration group, only three patients (11%) were readmitted for congestive HF (the primary endpoint), and all completed a fresh ultrafiltration session as required by the protocol. During the follow-up, only one of them required additional hospitalisation for congestive heart failure, and this patient received ultrafiltration once more as treatment. Consequently, during the one-year follow-up, the ultrafiltration group experienced 4 rehospitalisations for congestive HF. In contrast, 14 patients (48%) in the control group experienced at least one rehospitalisation for congestive HF; over the course of the one-year follow-up, this amounted to a total of 30 rehospitalisations in the control group. Although there was no statistically significant difference in the amount of body weight lost at discharge between the two treatment approaches, the trial demonstrated a decreased rate of rehospitalisation at one year in patients treated with UF in comparison to those treated with normal diuretic therapy [51].

The most recent randomised multi-centre study is AVOID-HF (aquapheresis versus intravenous diuretics and hospitalisation for heart failure). The patients in the UF group received UF for an average of 80-53 hours at a rate of 138-47 ml/h (range: 50-300 ml/h) during the index hospitalisation. The standard therapy patients received an average IV loading dosage of furosemide equivalent to 271.26263.06 mg (range 36 to 1,446.67 mg) for a typical 100- to 78-hour period. According to data from the AVOID-HF trial, the UF group had a non-statistically significant trend towards a longer time until the first HF event following the index hospitalisation, significantly fewer patients were rehospitalised for heart failure or cardiovascular reasons at 30 days, and shorter rehospitalisations for HF or cardiovascular reasons at 30 days compared to the diuretic group. The frequency of patients reporting an adverse event of particular interest or a major product-related side effect was higher in the UF group than in the diuretic group, despite the fact that 90-day mortality did not differ between the groups. Because the sponsor unilaterally and prematurely ended the study, the findings of the AVOID-HF trial should be regarded with caution. While the UF arm was found to have had fewer patients with HF rehospitalisations and a fewer number of patients with cardiovascular rehospitalisations, it seemed that the time to the first heart failure event after discharge was prolonged for the UF group compared to standard care [32].

## Conclusions

In view of the above, it is safe to say ultrafiltration is a safe and effective management strategy for heart failure patients. It has been demonstrated in a number of trials that UF eventually decreases the total number of heart failure-related hospitalisations. However, it is important that we generate precise algorithms for the selection criteria of the patients. Adverse effects related to UF, such as bleeding, clotting, and glomerular injury, should be further assessed and analysed thoroughly. A number of trials have also established the fact that UF does not contribute to electrolyte abnormalities. This can be further established by more analytically designed trials. The effects of this treatment on overall mortality need to be properly investigated in future studies. Given the ambit of unexplored and ambiguous areas of UF, it is of vital importance that there be an increase in government-funded clinical trials and research studies to establish UF as one of the major therapies for congestive heart failure patients.
